#  Two Cases of Neonatal Gastric Perforation 

**Published:** 2015-07-01

**Authors:** Abdullahi LB, Mohammad AM, Anyanwu LJC, Farinyaro AU

**Affiliations:** Pediatric Surgery Unit, Surgery Department, Aminu Kano Teaching Hospital/ Bayero University, Kano

**Dear Sir**

One-day-old term male newborn admitted with features of birth asphyxia. An NG tube was passed and IV fluids with parenteral antibiotics were started. Next day, the patient developed a progressive abdominal distention and bilious vomiting. A plain abdominal X-ray showed evidence of massive pneumoperitoneum. Laboratory tests were within normal limits. At operation, there was an ovoid gastric perforation measuring about 1cm in diameter on the lesser curvature of the stomach which was closed in 2 layers. The postoperative recovery was uneventful. 

Another 6-day-old male neonate presented with a 3 day history of fever and constipation. An initial diagnosis of neonatal sepsis was made, a nasogastric (NG) tube was passed, and parenteral antibiotics along with intravenous (IV) fluid were started. The patient developed progressive abdominal distension. Laboratory tests showed element of sepsis but blood culture did not yield any isolate. At operation, an oval perforation was found on the greater curvature of the stomach with the NG tube coming through the perforation into peritoneal cavity (Fig. 1). The perforation was closed in 2 layers. The patient developed jaundice with a high grade fever during the post-operative period and succumbed to sepsis.

The cause of gastric perforation is not overt often and can be traumatic, ischaemic, or spontaneous. In neonates, the circular muscle layer of the stomach normally contains several gaps, most prominently in the fundus, near the greater curvature that may have some role in gastric perforation. These gaps are more common in premature infants.[3] In our series however both of the patients were term neonates. Perforation of gastric stress ulcers have been reported in critically ill neonates. Ischemic gastric perforations have also been noted in conjunction with necrotizing enterocolitis.[2, 4] Iatrogenic trauma by vigorous nasogastric or orogastric tube placement causes perforation and appears as puncture wounds or short lacerations; these traumatic gastric perforations may develop during the course of positive pressure ventilation.[1] The perforation is more common along the greater curvature of the stomach as seen in one of our patients, though can occur anywhere. In our first patient, the more plausible cause of gastric perforation seemed a NG tube insertion; in second patient, though NG tube was inserted, but the presence of fever and signs of infection may point to the septic etiology.

**Figure:**
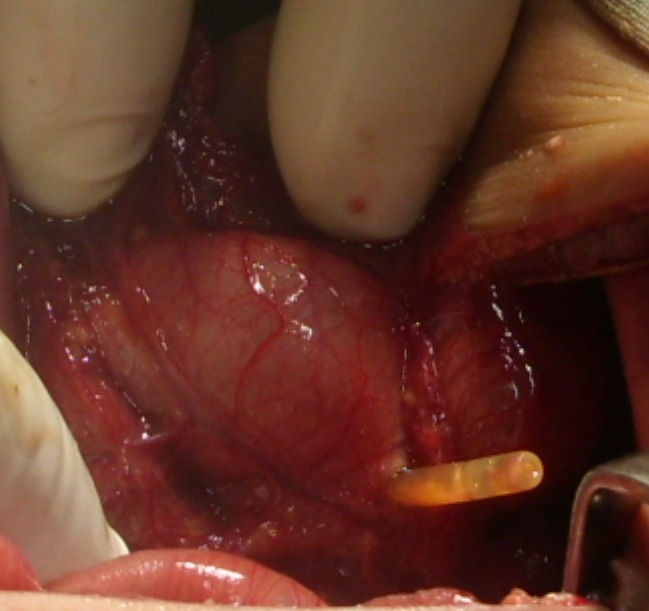
Figure1: Gastric perforation with NG tube coming out of it.

## Footnotes

**Source of Support:** Nil

**Conflict of Interest:** None

